# Overexpression of **α**(1,3)-fucosyltransferase VII is sufficient for the acquisition of lung colonization phenotype in human lung adenocarcinoma HAL-24Luc cells

**DOI:** 10.1038/sj.bjc.6690482

**Published:** 1999-06

**Authors:** M Martín-Satué, C de Castellarnau, J Blanco

**Affiliations:** Department Biologia Cellular, Institut de Recerca Oncològica, Autovia Castelldefels Km 2, Hospitalet de Llobregat, Barcelona, 08907, Spain; Laboratory of Atherothrombosis and Vascular Biology, Institut de Recerca, Hospital Santa Creu i Sant Pau, Barcelona, 08025, Spain; Centro de Investigación y Desarrollo, Consejo Superior de Investigaciones Científicas, Barcelona, 08028, Spain

**Keywords:** metastasis, sialyl-Lewis^x^, E-selectin, transfection, human lung adenocarcinoma

## Abstract

Metastatic human lung adenocarcinoma HAL-8Luc cells display an enhanced expression of α(1,3)-fucosyltransferases (α(1,3)-Fuc-Ts) compared with their non-metastatic counterpart HAL-24Luc cells. This correlates with an increased surface expression of Lewis^x^(Le^x^)- and Lewis^a^(Le^a^)-related molecules and an in vitro enhanced adhesive capacity to E-selectin-expressing endothelial cells (Martin-Satué et al (1998). *Cancer Res***58**: 1544–1550). In the present work we have stably transfected HAL-24Luc cells with the cDNAs for the α(1,3)-Fuc-TIV and VII enzymes and analysed by flow cytometry the expression of Le^x^, sialyl-Le^x^, sialyl-Le^x^dimeric, Le^a^and sialyl-Le^a^. Fuc-TVII transfectants exclusively overexpress sialyl-Le^x^while Fuc-TIV-transfected cells only overexpress the Le^x^oligosaccharide. We show that solely Fuc-TVII transfectants are able to adhere to interleukin-1β-stimulated HUVEC monolayers. We also demonstrate that Fuc-TVII overexpression in HAL-24Luc cells is sufficient for the acquisition of the lung colonization phenotype. This is the first report directly showing the contribution of an α(1,3)-Fuc-T to the metastatic behaviour of human lung adenocarcinoma cells. © 1999 Cancer Research Campaign


*British Journal of Cancer* (1999) **80**(8), 1169–1174
© 1999 Cancer Research Campaign
Article no. bjoc.1999.0482

### Overexpression of α(1,3)-fucosyltransferase VII is sufficient for the acquisition of lung colonization phenotype in human lung adenocarcinoma HAL-24Luc cells

**M Martín-Satué[1], C de Castellarnau[2] and J Blanco[1,3]**

[1]Department Biologia Cellular, Institut de Recerca Oncològica, Autovia Castelldefels Km 2,7, 08907 Hospitalet de Llobregat, Barcelona, Spain; [2]Laboratory of Atherothrombosis and Vascular Biology, Institut de Recerca, Hospital Santa Creu i Sant Pau, 08025 Barcelona, Spain; and [3]Centro de Investigación y Desarrollo, Consejo Superior de Investigaciones Científicas, 08028 Barcelona, Spain

**Summary** Metastatic human lung adenocarcinoma HAL-8Luc cells display an enhanced expression of α(1,3)-fucosyltransferases (α(1,3)-Fuc-Ts) compared with their non-metastatic counterpart HAL-24Luc cells. This correlates with an increased surface expression of Lewis$^x$ (Le$^x$)- and Lewis$^a$ (Le$^a$)-related molecules and an in vitro enhanced adhesive capacity to E-selectin-expressing endothelial cells (Martin-Satué et al (1998). *Cancer Res* **58**: 1544–1550). In the present work we have stably transfected HAL-24Luc cells with the cDNAs for the α(1,3)-Fuc-TIV and VII enzymes and analysed by flow cytometry the expression of Le$^x$, sialyl-Le$^x$, sialyl-Le$^x$ dimeric, Le$^a$ and sialyl-Le$^a$. Fuc-TVII transfectants exclusively overexpress sialyl-Le$^x$ while Fuc-TIV-transfected cells only overexpress the Le$^x$ oligosaccharide. We show that solely Fuc-TVII transfectants are able to adhere to interleukin-1β-stimulated HUVEC monolayers. We also demonstrate that Fuc-TVII overexpression in HAL-24Luc cells is sufficient for the acquisition of the lung colonization phenotype. This is the first report directly showing the contribution of an α(1,3)-Fuc-T to the metastatic behaviour of human lung adenocarcinoma cells.

**Keywords**: metastasis; sialyl-Lewis$^x$; E-selectin; transfection; human lung adenocarcinoma

Lung cancer is the most prevalent cause of cancer death in most countries. In several areas the annual incidence rates among males exceed 100 per 100 000, some of the highest reported for any malignancy (Blot et al, 1996). Adenocarcinoma, which arises from glandular epithelia, is a major form of this pathology. Metastatic spread of primary tumour cells is the main cause of death in oncological patients. Aberrant cell surface glycosylation plays an important role on tumour cell adhesion to endothelium, a necessary event leading to extravasation into target organs (Hakomori, 1989).

Carcinoma cells are often enriched with sialylated fucosylated lactosaminoglycans such as sialyl-Lewis$^x$ (sialyl-Le$^x$) and sialyl-Lewis$^a$ (sialyl-Le$^a$), which are recognized by the endothelial cell adhesion molecule E-selectin (Takada et al, 1993; Sawada et al, 1994; Yamada et al, 1997). It has been proposed that E-selectin promotes attachment of tumour cells to endothelium by molecular interactions similar to those used in leukocyte recruitment to inflammation sites (Bevilacqua and Nelson, 1993; Varki, 1994; Lowe, 1997). The final step in the synthesis of these side-chain oligosaccharides is dependent on the activity of one or more specific α-(1,3)-fucosyltransferases (α-(1,3)-Fuc-Ts), and to date, five *α-(1,3)-Fuc-T* genes have been cloned: *Fuc-TIII* (Kukowska-Latallo et al, 1990), *Fuc-TIV* (Goelz et al, 1990; Lowe et al, 1991), *Fuc-TV* (Weston et al, 1992*a*), *Fuc-TVI* (Weston et al, 1992*b*) and *Fuc-TVII* (Natsuka et al, 1994; Sasaki et al, 1994). These enzymes catalyse the transfer of fucose to type 2 chain-based structures (Galβ1–4GlcNAc–R) to generate Le$^x$-related molecules and Fuc-TIII (the Lewis type enzyme) can also efficiently use type 1 carbohydrates (Galβ1–3GlcNAc–R) to yield Le$^a$ and sialyl-Le$^a$ determinants. Transfection studies have shown that the five known α-(1,3)-Fuc-T enzymes are able to synthesize the sialyl-Le$^x$ determinant, although the role of Fuc-TIV, the myeloid type enzyme, in this synthesis has only been reported for few cell types such as a CHO-derived cell line (Goelz et al, 1994). This enzyme has, however, high affinity for non-sialylated type 2 acceptor and thus participates on the synthesis of Lewis$^x$ antigen. Fuc-TV and Fuc-TVI (the plasma type enzyme) have the ability to synthesize Le$^x$ and sialyl-Le$^x$ (Weston et al, 1992*a*, 1992*b*). Fuc-TVII, a leukocyte-expressed enzyme, is unique in that this enzyme can synthesize only sialy-Le$^x$ (Natsuka et al, 1994; Sasaki et al, 1994). Several reports have already confirmed the key role of this enzyme in the control of leukocyte trafficking in health and disease (Hiraiwa et al, 1996; Malý et al, 1996). Expression of Fuc-TIV and Fuc-TVII genes had initially been related to poor prognosis in lung cancers (Ogawa et al, 1996) but, more recently, Fuc-TVII has been defined as a more important indicator of a poor prognosis by its participation in sialyl-Le$^x$ synthesis (Ogawa et al, 1997).

We have previously demonstrated that the metastatic human lung adenocarcinoma cell line HAL-8Luc overexpressed a series of sialylated fucosylated lactosaminoglycans compared to the non-metastatic HAL-24Luc cells (Martín-Satué et al, 1998). Both cell lines were derived from the same common parental cell line KUM-LK-2 using repeated cloning with limiting dilution technique and intravenous (i.v.) injection in *nu*/*nu* mice (Inufusa et al,

*Received 6 October 1998*
*Revised 5 January 1999*
*Accepted 6 January 1999*

*Correspondence to:* J Blanco

1991). Our previous characterization of these cells indicated that the lungs are the only organs colonized when HAL-8Luc cells were inoculated either i.v. or intramuscularly (i.m.) in athymic mice while HAL-24Luc cells did not metastasize. Overexpression of these oligosaccharides correlated with an increased expression of the five *α-(1,3)-Fuc-T* genes and enhanced adhesion to activated endothelial cells. Results from those in vitro adhesion assays pointed to the relevance of the sialyl-$Le^x$ oligosaccharide in the binding reaction and indicated a high affinity of this epitope for E-selectin expressed on endothelial cells. These observations reinforced previous findings in which sialyl-$Le^x$ had been identified as predicting factor of recurrence in lung adenocarcinoma patients (Ogawa et al, 1994). The aim of the present work was to analyse if α(1,3)-Fuc-TIV and α(1,3)-Fuc-TVII overexpression modifies the adhesive properties and lung colonization potential of non-metastatic human lung adenocarcinoma HAL-24Luc cells.

### MATERIALS AND METHODS

### Cell lines

Human lung adenocarcinoma cell lines HAL-8Luc (metastatic) and HAL-24Luc (non-metastatic) (Martín-Satué et al, 1998) were maintained in RPMI-1640 (Bio-Whitaker, Verviers, Belgium) supplemented with 10% (v/v) heat-inactivated fetal bovine serum (FBS) (Bio-Whitaker), 2 mM L-glutamine (Bio-Whitaker), penicillin (100 units $ml^{-1}$), streptomycin (100 μg $ml^{-1}$) and 300 μg $ml^{-1}$ geneticin (G418; Life Technologies, Inc., Grand Island, NY, USA) in a humidified atmosphere of 5% carbon dioxide at 37°C.

Human umbilical vein endothelial cells (HUVECs) were isolated by collagenase digestion from multiple segments of normal-term umbilical cords and cultured as previously described (López et al, 1993). Adhesion assays were performed using endothelial cells within three passages.

### Plasmids and transfection

Plasmids containing full-length cDNAs for Fuc-TIV (pcDNAI-α(1,3)$FT_{Mlu}$) and Fuc-TVII (pCDM8-FucTVII) were kindly donated by Dr John B Lowe from the Howard Hughes Medical Institute (The University of Michigan Medical Centre, Ann Arbor, MI, USA). Plasmid pCEP4 encoding hygromycin resistance as well as the pcDNAI and pCDM8 plasmids were obtained from Invitrogen (San Diego, CA, USA).

HAL-24Luc cells, which were already neomycin resistants, were co-transfected by lipofection with each Fuc-T cDNA-containing plasmid together with the pCEP4 plasmid, using the method supplied by the manufacturer (Life Technologies, Inc., Grand Island, NY, USA). HAL-24Luc cells mock transfected with pCEP4 and the vector devoid of Fuc-T cDNA were used as control clones. Transfected cells were selected by supplementing culture medium with 100 μg $ml^{-1}$ hygromycin (Boehringer Mannheim, Mannheim, Germany). After limiting dilution, culture-resistant clones were analysed for surface antigens expression.

### Flow cytometry

Up to 25 HAL-24Luc clones from each transfection were analysed for cell surface antigen expression by flow cytometric analysis (Epics XL-MCL, Hialeah, FL, USA) as previously described (Martín-Satué et al, 1998) using the following primary monoclonal antibodies (mAbs): FH6, an IgM anti-sialyl-$Le^x$ dimeric, kindly provided by Otsuka Pharmaceutical (Osaka, Japan); CSLEX-1, an IgM anti-sialyl-$Le^x$ and Leu-M1, an IgM anti-$Le^x$ both purchased from Becton Dickinson (San Jose, CA, USA); LE-1, an IgM anti-$Le^a$ (Ortho Diagnostic, Neckargemünd, Germany); CA19.9, an $IgG_1$ anti-sialyl-$Le^a$ (Novocastra Laboratory Ltd, Newcastle, UK). These specific antibodies were used at saturating concentrations determined by titration.

Cells labelled with irrelevant isotypic murine mAbs (mouse anti-human $IgG_1$ and mouse anti-human IgM from Becton Dickinson, San Jose, CA, USA) or secondary antibody alone (fluorescein-conjugated goat anti-mouse IgG; DAKO, A/S, Copenhagen, Denmark) were used as controls.

Mean channels of fluorescence were recorded for every analysis, and the ratios of mean channels of fluorescence for each mAb and control pair were also calculated.

### Northern blot

Four micrograms poly-adenosine$^+$ (poly-$A^+$) RNA from transfected and untransfected HAL-24Luc cells were fractionated by formaldehyde/agarose gel electrophoresis and transferred to nylon membranes (Hybond-$N^+$; Amersham International, Buckinghamshire, UK). The membranes were hybridized with $^{32}$P-labelled cDNA probes in a solution containing 50% (v/v) formamide, 5× saline–sodium citrate (SSC), 50 mM sodium phosphate buffer pH 6.5, 250 μg $ml^{-1}$ sheared salmon sperm DNA, 10 × Denhart's solution and 10% dextran sulphate overnight at 42°C. Following hybridization the membranes were washed twice for 10 min at room temperature in 2 × SSC, 0.1% sodium dodecyl sulphate (SDS) and once in 0.2 × SSC, 0.1% SDS for 1 h at 68°C. Finally, the membranes were autoradiographed by exposure to Hyperfilm MP (Amersham International, Buckinghamshire, UK). Blots were stripped and reprobed with human β-actin cDNA as a control for RNA integrity and loading consistency. Band intensity was analysed with the Molecular Analyst/PC 1.4 software (BioRad, Hercules, CA, USA) and calibrated by comparison with the β-actin bands. Fuc-Ts and β-actin human cDNA probes were obtained in our laboratory (Martín-Satué et al, 1998).

### Endothelial cell adhesion assays

HUVECs were cultured in fibronectin-coated 96-well microtitre plates. Confluent endothelial cells were induced for E-selectin expression by incubating with 10 U $ml^{-1}$ human recombinant interleukin (IL)-1β (Boehringer Mannheim, Mannheim, Germany) for 4 h at 37°C previous to the experiment.

HAL-8Luc, HAL-24Luc and Fuc-T-transfected HAL-24Luc cells were detached by trypsinization, labelled with 200 μCi of sodium [$^{51}$Cr] chromate (Amersham International, Amersham, UK) and added in RPMI-1640 with 2% FBS to both stimulated and non-stimulated HUVEC monolayers at a nominal density of $1 \times 10^5$ per well. After a 30 min incubation period at 4°C non-adherent cells were removed by aspiration and following two washes with 2% FBS medium bound cells were lysed with 25 mM sodium hydroxide, 0.1% SDS and radioactivity was determined using a γ-counter (1261 Multigamma; WALLAC Oy, Türku, Finland). Adhesion percentages were calculated as the ratio of bound cell radioactivity to total radioactivity from $1 \times 10^5$ cells.

For E-selectin blockade, IL-1β-stimulated HUVEC monolayers were incubated with 10 μg $ml^{-1}$ anti-human E-selectin mAb, P2H3

 *© 1999 Cancer Research Campaign*

(Chemicon International Inc., Temecula, CA, USA) for 30 min at 4°C previous to the binding assay.

For inhibition assays, labelled tumour cells were first incubated with the above described mAbs: Leu-M1, CSLEX-1, FH6, LE-1 or CA19.9 at a saturating concentration of 50 μg $ml^{-1}$ for 30 min at room temperature and then added to HUVEC monolayers. Isotypic irrelevant murine mAbs (mouse anti-human $IgG_1$ and mouse anti-human IgM from Becton Dickinson, San Jose, CA, USA) were used as controls.

### Experimental metastasis assay

Metastasis assays were performed on 7-week-old female BALB/c homozygous nude (*nu/nu*) mice (CRIFFA S.A., Barcelona, Spain) maintained in a specific pathogen-free environment.

The above described HAL-24Luc Fuc-T-transfectants ($5 \times 10^5$ cells) suspended in 0.1 ml serum-free RPMI-1640 were injected in the lateral tail vein to assess for lung colonization potential. Untransfected HAL-8Luc and HAL-24Luc cells as well as mock-transfectants were also inoculated as controls in these experiments. Assays were done in duplicate and five animals were used for each replica. Animals were sacrified 10 weeks after injections. No animals died before the time of sacrifice. Following post-mortem examination, lungs were collected for histopathological analysis.

Samples were fixed in 4% (w/v) paraformaldehyde in 0.1 M PBS for 2 h at room temperature, dehydrated and paraffin-embedded. Nominal 6-μm-thick sections were mounted on glass slides and routinely stained with haematoxylin and eosin. Analysis of these samples was performed following 'systematic random sampling' methodology (Pakkenberg and Gundersen, 1988). Specimens were observed and photographed with a Reichert-Jung Polyvar2 light microscope.

### RESULTS

### Flow cytometry

Fuc-T-transfected HAL-24Luc cell clones were examined by flow cytometry for surface expression of the following fucosylated lactosaminoglycans: $Le^x$, sialyl-$Le^x$, sialyl-$Le^x$ dimeric, $Le^a$ and sialyl-$Le^a$.

Fuc-TIV transfectants only overexpressed $Le^x$ while Fuc-TVII-transfected cells only showed enhanced expression of sialyl-$Le^x$ oligosaccharide. The highest antigen expressing clone from Fuc-TIV and Fuc-TVII transfections were named 24FT4 and 24FT7, respectively, and selected for experiments. The ratios of mean fluorescence intensity were 172 for $Le^x$ in 24FT4 cells and 48 for sialyl-$Le^x$ in 24FT7 cells. No significant increases in $Le^a$, sialyl-$Le^a$ or sialyl-$Le^x$ dimeric expression were detected in any of the clones analysed. Mock transfectants displayed the same antigenic pattern as untransfected cells indicating that the transfection process did not modify the constitutive expression of these oligosaccharides. Cytofluorometric results are illustrated in Figure 1.

### Northern blot analysis

All the Fuc-T transfectants expressed higher mRNA levels for the analysed Fuc-Ts than the parental HAL-24Luc cells or the mock transfectants (Figure 2). Mock transfectants expressed amounts of Fuc-TIV mRNA equivalent to those of HAL-24Luc cells, showing

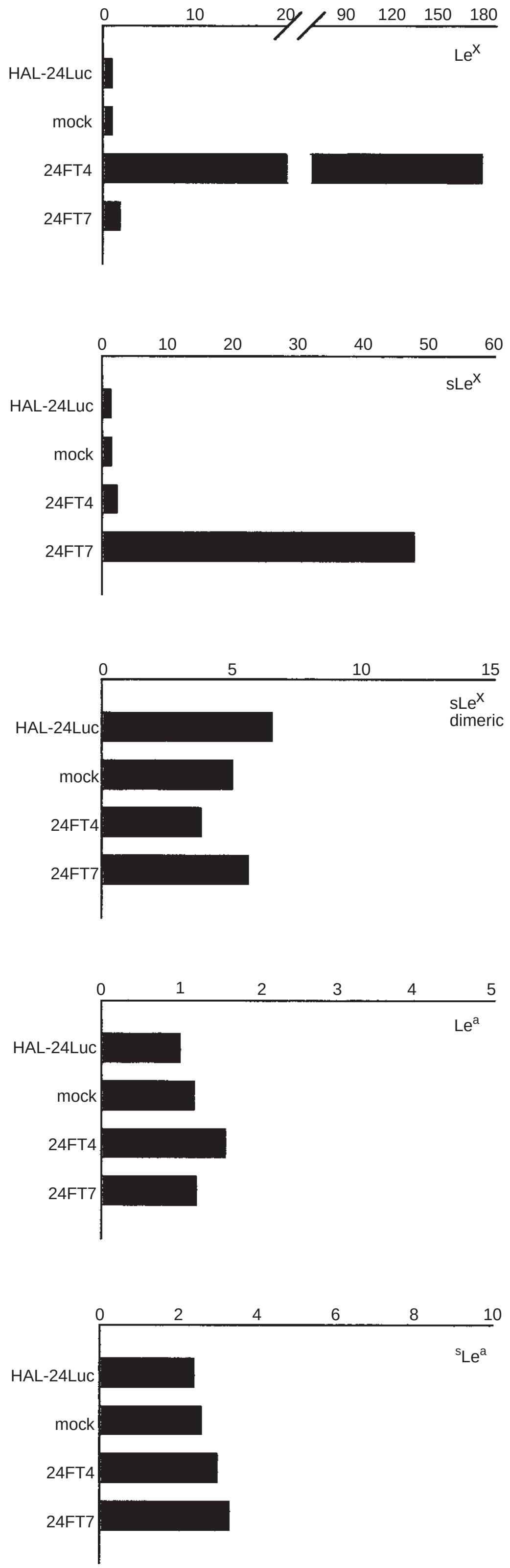

**Figure 1** Flow cytometry analysis of HAL-24Luc cells, mock-transfectants and 24FT4 and 24FT7 clones. Cells were labelled with mAb against $Le^x$ (Leu-M1 mAb), sialyl-$Le^x$ (CSLEX-1 mAb), sialyl-$Le^x$ dimeric (FH6 mAb), $Le^a$ (LE-1 mAb) and sialyl-$Le^a$ (CA19.9 mAb). Bars represent the ratio of mean fluorescence intensities between each mAb and its control. Data correspond to one representative of five similar experiments. Notice difference in the ordinate scales

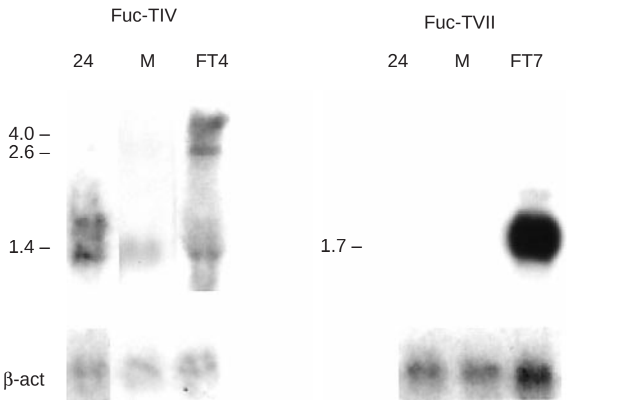

**Figure 2** Northern blot analysis of mRNA from HAL-24Luc (*24*), mock-transfected (*M*) and Fuc-T-transfected (*FT4, FT7*) cells. Blots were hybridized with the $^{32}$P-labelled Fuc-TIV and Fuc-TVII cDNA probes as described in 'Materials and Methods'. After a first autoradiographic exposure, the probes were stripped out and the blots were rehybridized with the β-actin probe (*β-act*). Band sizes are indicated in kilobases at the *left* of each set that co-transfections did not affect the constitutive Fuc-TIV expression on these cells. Mock transfectants as well as HAL-24Luc cells showed undetectable levels of Fuc-TVII mRNA.

### Endothelial cell adhesion assays

We have studied the relative ability of Fuc-T-transfected HAL-24Luc cell lines, 24FT4 and 24FT7, to adhere to IL-1β-stimulated HUVEC monolayers. Mock transfected HAL-24Luc cells as well as metastatic HAL-8Luc cells were also subjected to adhesion assays and used as controls. Since we have previously shown that HAL-8Luc cells significantly adhere to E-selectin expressing HUVEC monolayers (Martín-Satué et al, 1998) we have taken the amount of HAL-8Luc binding in these adhesion assays as 100%. Normalized values for the mean and standard deviation (s.d.) of these binding results are illustrated in Figure 3.

The sialyl-Le$^x$-overexpressing cells 24FT7 showed extensive adhesion to IL-1β-stimulated HUVEC monolayers, but did not detectably bind to non-activated endothelial cells. On the contrary, 24FT4 cells were unable to bind to HUVEC cells regardless of their activation state (Figure 3A). None of the mock-transfectant clones analysed was able to adhere to endothelial cells, indicating that transfection process by itself did not alter the adhesive properties of HAL-24Luc cells.

Blocking of sialyl-Le$^x$ oligosaccharides with the CSLEX-1 mAb resulted in 90% inhibition of binding in Fuc-TVII-transfected cells (Figure 3B), demonstrating the key role of this determinant in the adhesion. Moreover, pretreatment of stimulated HUVEC monolayers with anti-E-selectin mAb completely abolished the binding of tumour cells. Irrelevant mAbs used as controls as well as other anti-fucosylated antigen mAbs tested had no effect on adhesion.

### Experimental metastasis assays

To analyse if Fuc-T overexpression in HAL-24Luc cells modifies their lung colonization potential, we have i.v. inoculated athymic mice with 3 Le$^x$-overexpressing Fuc-TIV-transfected HAL-24Luc clones, including the 24FT4 and 4 sialyl-Le$^x$-overexpressing

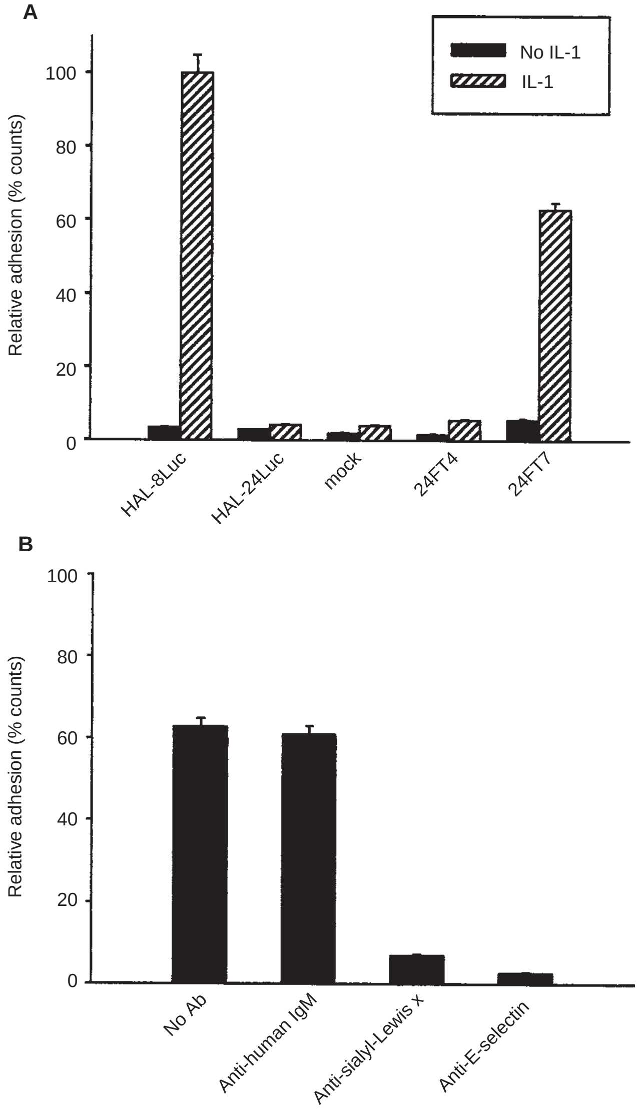

**Figure 3** Adhesion of tumour cells to HUVEC monolayers. Data show normalized values corresponding to the fraction of bound cells and represent the means of four different experiments, each performed in six replicates. (**A**) Adhesion of: HAL-8Luc, HAL-24Luc, mock-transfected HAL-24Luc, 24FT4 and 24FT7 cells to non-stimulated (solid bars) or IL-1 β-stimulated (striped bars) HUVECs. (**B**) Adhesion of 24FT7 cells to IL-1-stimulated HUVECs after no treatment, pretreatment of tumour cells with an irrelevant mAb (anti-human IgM), anti-sialyl-Le$^x$ mAb or pretreatment of HUVECs with anti-E-selectin mAb

**Table 1** Production of lung metastasis by tumour cells i.v. injected in athymic mice. Entries in bold indicate the clones whose analysis is described

| Cell line | Number of mice with lung metastasis/total | Total number of lung colonies/mice |
|---|---|---|
| HAL-8Luc | 9/10 | **8–25** |
| HAL-24Luc | 0/10 | 0 |
| Mock-transfected HAL-24Luc | 0/10 | 0 |
| **24FT4** | **0/10** | **0** |
| 24FT4b | 0/5 | 0 |
| 24FT4c | 0/5 | 0 |
| **24FT7** | **8/10** | **4–30** |
| 24FT7b | 4/5 | 5–20 |
| 24FT7c | 4/5 | 10–25 |
| 24FT7d | 3/5 | 7–20 |

 *© 1999 Cancer Research Campaign*

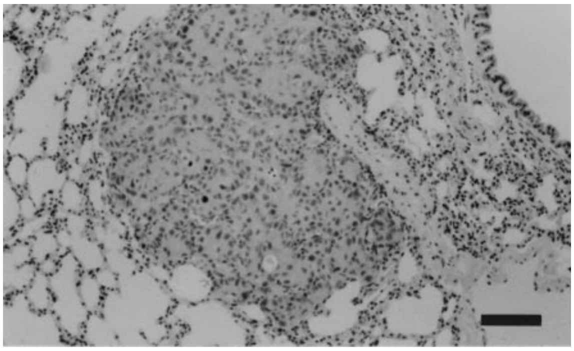

**Figure 4** Light micrograph of lung metastasis developed by 24FT7-inoculated mouse showing a tightly packed tumour cell mass partially occupying the aerial spaces. *bar*, 100 µm

Fuc-TVII-transfected HAL-24Luc clones, including the 24FT7. Untransfected HAL-8Luc, HAL-24Luc cells and HAL-24Luc mock transfectants were also inoculated and used as controls in these experiments.

Seventy-six per cent (19 of 25) mice i.v. inoculated with Fuc-TVII-overexpressing HAL-24Luc cells developed lung metastases. Similarly, 90% (nine of ten) mice i.v. inoculated with HAL-8Luc cells and used as positive controls for the inoculation procedure, developed pulmonary colonies. No metastases were detected in mice i.v. inoculated with either untransfected, mock-transfected or Fuc-TIV-overexpressing HAL-24Luc cells. These results are summarized in Table 1.

Metastases derived from mice i.v. inoculated with Fuc-TVII-overexpressing HAL-24Luc cells were associated with the basal lamina of the terminal bronchiole epithelium as was also previously described for the HAL-8Luc-inoculated mice (Martín-Satué et al, 1998). A typical lung metastasis from 24FT7-inoculated mouse is shown in Figure 4.

### DISCUSSION

Side-chain oligosaccharides such as sialyl-$Le^x$ and sialyl-$Le^a$ in both glycolipids and glycoproteins on the surface of tumour cells had already been identified as tumour-associated antigens (Hakomori, 1989). More recently, these epitopes have also been characterized as E- and P-selectin counter-receptors expressed in several leukocyte subsets and participate in the recruitment of leukocytes during inflammation (Varki, 1994). Similar mechanisms have been proposed to participate in the metastatic spread of primary tumour cells. It has been suggested that P-selectin may mediate the formation of large aggregates of tumour cells and platelets that could be retained in the thin capillaries of the lung promoting growth and organ colonization (Kim et al, 1998). Previous reports have shown that sialyl-$Le^x$ expression might be a useful indicator of blood vessel invasion and recurrence in lung adenocarcinoma (Ogawa et al, 1994). We have also demonstrated that enhanced surface display of sialyl-$Le^x$ on human lung adenocarcinoma cells correlated with their metastatic ability and participated in the adhesion of tumour cells to E-selectin-expressing endothelial cells (Martín-Satué et al, 1998). Expression of α-(1,3)-Fuc-TVII gene has also been related to poor prognosis in lung cancer because of its participation in sialyl-$Le^x$ synthesis (Ogawa et al, 1997). To determine the role of Fuc-TIV and Fuc-TVII in the metastatic behaviour of lung adenocarcinoma cells we have overexpressed these enzymes in the non-metastatic HAL-24Luc cells.

In the present work, we have stably transfected the non-metastatic human lung adenocarcinoma HAL-24Luc cell line with cDNAs encoding the α-(1,3)-Fuc-TIV and VII enzymes and analysed the resulting changes in antigenic expression, adhesive ability and metastatic potential of the transfected cell lines. Our cytofluorometric results indicated that 24FT4 and 24FT7 cells exclusively overexpressed $Le^x$ and sialyl-$Le^x$ determinants respectively. FH6 mAb did not detect any increase in sialyl-$Le^x$ dimeric antigen expression. Neither $Le^a$ nor sialyl-$Le^a$ had been expressed in any of the clones analysed, and both showed the same background expression level as the parental HAL-24Luc cells confirming that neither of these enzymes recognize type 1 chain oligosaccharides. The pattern of Lewis-related antigens displayed by the transfectants studied in this work is in accordance with that from previously reported transfection experiments in which Fuc-Ts were overexpressed in other cell lines such as CHO or leukocyte-derived cells (Natsuka et al, 1994; Sasaki et al, 1994). Synthesis of sialyl-$Le^x$ involves sialylation of a type 2 lactosamine core (Galβ1–4GlcNAc–R) followed by the addition of one fucosyl residue. Untransfected HAL-24Luc cells did not produce sialyl-$Le^x$ but Fuc-TVII transfection of these cells resulted in sialyl-$Le^x$ overexpression, indicating that the sialylated intermediates are not the limiting molecules and pointing to the relevance of Fuc-TVII in this reaction.

Endothelial cell adhesion experiments performed with the selected Fuc-T transfectants showed that only sialyl-$Le^x$-overexpressing HAL-24Luc cells (24FT7 clone) had the ability to bind to IL-1-stimulated HUVECs in contrast with the null adhesion capacity displayed by 24FT4, mock-transfected or untransfected cells. Binding inhibition achieved either by blocking sialyl-$Le^x$ on the tumour cells or by E-selection blockade on HUVECs emphasized the key role of these molecules in the binding reaction.

By inoculation of transfected cells in athymic mice we demonstrated that Fuc-TVII overexpression is sufficient for acquisition of lung colonization phenotype by HAL-24Luc cells. The absence of metastatic foci in 24FT4-inoculated mice is likely related to the null in vitro E-selectin adhesion capacity shown by these cells. Our experiments strongly suggest the participation of the endothelial E-selectin in the initial stages of lung colonization by the HAL adenocarcinoma cells. Although in a different cell system, the existence of a mechanism similar to that described by Kim et al (1998) contributing to colonization can not be excluded. Furthermore, it may help explain organ selectivity.

The fact that the sole Fuc-TVII transfection rendered the HAL-24Luc cells metastatic emphasizes the convenience in using models consisting of genetically closely related cell lines such as the HAL-8Luc/HAL-24Luc for this work. Our results not only reinforce previous findings in which Fuc-TVII expression has been reported as an indicator of poor prognosis in lung carcinoma (Ogawa et al, 1996, 1997), but also directly demonstrate for the first time the role of this enzyme on the acquisition of lung colonization behaviour by human lung adenocarcinoma cells.

### ACKNOWLEDGEMENTS

We thank Dr John B Lowe (Howard Hughes Medical Institute, The University of Michigan Medical Centre, Ann Arbor, MI, USA) for the kind donation of the plasmids containing cDNAs for the five Fuc-T enzymes used in this work; Dr O Matsuo (Kinki University

School of Medicine, Osakasayame City, Japan) for providing the HAL-8 and HAL-24 cell lines; Dr Sen-itiroh Hakomori (Pacific Northwest Research Foundation, University of Washington, WA, USA) for help in acquiring FH6 mAb; Otsuka Pharmaceutical (Osaka, Japan) for the FH6 mAb gift and Dr J García-Valero (Departamento Biologia Cellular, Facultat de Biologia, Universitat de Barcelona, Barcelona, Spain) for valuable support in histopathological analysis and critical review of the manuscript. We also thank Rosabel Marrugat (Institut Recerca Oncològica, Barcelona, Spain), Almudena García (Serveis Científico-Tècnics, Universitat de Barcelona, Barcelona, Spain), Dr Isabel Pich (Hospital de Sant Pau, Barcelona, Spain) and Blanca Luena (Animal Facilities, Institut de Recerca Oncològica, Barcelona, Spain) for excellent technical assistance. This work has been supported by FIS (Fondo de Investigaciones Sanitarias) SAF 97/0115 grant and funds from Servei Català de la Salut, Generalitat de Catalunya.

### REFERENCES

Bevilacqua MP and Nelson RM (1993) Selectins. *J Clin Invest* **91**: 379–387

Blot WJ and Fraumeni JF Jr (1996) Cancers of the lung and pleura. In: *Cancer Epidemiology and Prevention*, Schottenfeld D and Fraumeni JF Jr (Eds) pp. 637–665. Oxford University Press: New York

Goelz SE, Hession C, Goff D, Griffiths B, Tizard R, Newman B, Chi-Rosso G and Lobb R (1990) ELFT: a gene that directs the expression of an ELAM-1 ligand. *Cell* **63**: 1349–1356

Goelz S, Kumar R, Potvin B, Sundaram S, Brickelmaier M and Stanley P (1994) Differential expression of an E-selectin ligand (SLex) by two Chinese hamster ovary cell lines transfected with the same alpha (1,3)-fucosyltransferase gene (ELFT). *J Biol Chem* **269**: 1033–1040

Hakomori S (1989) Aberrant glycosylation in tumors and tumor-associated carbohydrate antigens. *Cancer Res* **52**: 257–331

Hiraiwa N, Dohi T, Kawakami-Kimura N, Yumen M, Ohmori K, Maeda M and Kannagi R (1996) Suppression of sialyl lewis x expression and E-selectin-mediated cell adhesion in cultured human lymphoid cells by transfection of antisense cDNA and of α1-3 fucosyltransferase (Fuc-TVII). *J Biol Chem* **271**: 31556–31561

Inufusa H, Kojima N, Yasutomi M and Hakomori S (1991) Human lung adenocarcinoma cell lines with different lung colonization potential (LCP), and a correlation between expression of sialosyl dimeric $Le^x$ (defined by MAb FH6) and LCP. *Clin Exp Metastasis* **9**: 245–257

Kim YJ, Borsig L, Varki NM and Varki A (1998) P-selectin deficiency attenuates tumor growth and metastasis. *Proc Natl Acad Sci USA* **95**: 9325–9330

Kukowska-Latallo JF, Larsen RD, Nair RP and Lowe JB (1990) A cloned human cDNA determines expression of a mouse stage-specific embryonic antigen and the Lewis blood group α (1,3/1,4) fucosyltransferase. *Genes Dev* **4**: 1288–1303

López S, Vila L, Breviario F and de Castellarnau C (1993) Interleukin-1 increases 15-hydroxyeicosatetraenoic acid formation in cultured human endothelial cells. *Biochim Biophys Acta* **1170**: 17–24

Lowe JB (1997) Selectin ligands, leukocyte trafficking, and fucosyltransferase genes. *Kidney Int* **51**: 1418–1426

Lowe JB, Kukowska-Latallo JF, Nair RP, Larsen RD, Marks RM, Macher BA, Kelly RJ and Ernst LK (1991) Molecular cloning of a human fucosyltransferase gene that determines expression of the Lewis x and VIM-2 epitopes but not ELAM-1-dependent cell adhesion. *J Biol Chem* **266**: 17467–17477

Malý P, Thall AD, Petryniak B, Rogers CE, Smith PL, Marks RM, Kelly RJ, Gersten KM, Cheng G, Saunders TL, Camper SA, Camphausen RT, Sullivan FX, Isogai Y, Hindsgaul O, von Adrial UH and Lowe JB (1996) The α(1,3)-fucosyltransferase Fuc-TVII controls leukocyte trafficking through an essential role in L-, E-, and P-selectin ligand biosynthesis. *Cell* **89**: 643–653

Martín-Satué M, Marrugat R, Cancelas JA and Blanco J (1998) Enhanced expression of α(1,3)-fucosyltransferase genes correlates with E-selectin-mediated adhesion and metastatic potential of human lung adenocarcinoma cells. *Cancer Res* **58**: 1544–1550

Natsuka S, Gersten KM, Zenita K, Kannagi R and Lowe JB (1994) Molecular cloning of a cDNA encoding a novel human leukocyte α-1,3-fucosyltransferase capable of synthesizing the sialyl lewis x determinant. *J Biol Chem* **269**: 16789–16794

Ogawa J, Sano A, Koide S and Shohtsu A (1994) Relation between recurrence and expression of proliferating cell nuclear antigen, sialyl $Lewis^x$, and sialyl $Lewis^a$ in lung cancer. *J Thorac Cardiovasc Surg* **108**: 329–336

Ogawa J, Inoue H and Koide S (1996) Expression of α-1,3-fucosyltransferase type IV and VII genes is related to poor prognosis in lung cancer. *Cancer Res* **56**: 325–329

Ogawa J, Inoue H and Koide S (1997) α-2,3-sialyltransferase type 3N and α-1,3-fucosyltransferase type VII are related to sialyl $Lewis^x$ synthesis and patient survival from lung carcinoma. *Cancer* **79**: 1678–1685

Pakkenberg B and Gundersen H (1988) Total number of neurons and glial cells in human brain nuclei estimated by dissector and the fractionator. *J Microsc* **150**: 1–20

Sasaki K, Kurata K, Funayama K, Nagata M, Watanabe E, Ohta S, Hanai N and Nishi T (1994) Expression cloning of a novel α1,3-fucosyltransferase that is involved in biosynthesis of the sialyl Lewis x carbohydrate determinants in leukocytes. *J Biol Chem* **269**: 14730–14737

Sawada R, Tsuboi S and Fukuda M (1994) Differential E-selectin-dependent adhesion efficiency in sublines of a human colon cancer exhibiting distinct metastatic potentials. *J Biol Chem* **269**: 1425–1431

Takada A, Ohmori K, Yoneda T, Tsuyuoka K, Hasegawa A, Kiso M and Kannagi R (1993) Contribution of carbohydrate antigens sialyl Lewis A and sialyl Lewis X to adhesion of human cancer cells to vascular endothelium. *Cancer Res* **53**: 354–361

Varki A (1994) Selectin ligands. *Proc Natl Acad Sci* **91**: 7390–7397

Weston BW, Nair RP, Larsen RD and Lowe JB (1992*a*) Isolation of a novel human α(1,3)fucosyltransferase gene and molecular comparison to the human lewis blood group α(1,3/1,4)fucosyltransferase gene. *J Biol Chem* **267**: 4152–4160

Weston BW, Smith PL, Kelly RJ and Lowe JB (1992*b*) Molecular cloning of a fourth member of a human α(1,3)fucosyltransferase gene family. *J Biol Chem* **267**: 24575–24584

Yamada N, Chung Y-S, Takasuka S, Arimoto Y, Sawada T, Dohi T and Sowa M (1997) Increased sialyl Lewis A expression and fucosyltransferase activity with acquisition of a high metastatic capacity in a colon cancer cell line. *Br J Cancer* **76**: 582–587

 *© 1999 Cancer Research Campaign*
